# Suicidal behaviours and their correlates in school-going Lebanese adolescents: findings from a national survey

**DOI:** 10.1186/s13034-023-00642-7

**Published:** 2023-07-12

**Authors:** Omid Dadras, Chia-Wen Wang

**Affiliations:** 1 Department of Global Public Health and Primary Care, University of Bergen, Bergen, Norway; 2 Section Global Health and Rehabilitation, Western Norway University of Applied Sciences, Bergen, Norway; 3 Saw Swee Hock School of Public Health, National University of Singapore and National University Health System, Singapore, Singapore; 4 Lloyd’s Register Foundation Institute for the Public Understanding of Risk, National University of Singapore, Singapore, Singapore

**Keywords:** Suicide, Adolescent, Substance use, Bullying, Physical harm, Mental health

## Abstract

**Background:**

Adolescent suicide is regarded as a serious phenomenon that affects the well-being of the youth. This study aims to investigate the prevalence of suicidal behaviours and their association with physical/psychological harm and substance use in a nationally representative sample of adolescents in Grades 7–12 in Lebanon.

**Methods:**

Data from the latest Global School-Based Student Health Survey conducted in 2017 were used in this study. The prevalence and correlates of suicidal ideation and suicide attempts among those who had considered committing suicide, at least once, were explored.

**Results:**

An estimated 13.45% of Lebanese adolescents, particularly females in Grades 7–12 had considered suicide at least once in the past 12 months. More importantly, almost half of them had attempted it at least once in the past 12 months. Those who frequently felt lonely or worried (previous year), were involved in physical fights or assaults (previous year), had been verbally or physically bullied (previous month), had missed more school days, experienced food insecurity, and had a history of substance abuse (marijuana, amphetamine, alcohol, and tobacco products) were more likely to exhibit suicidal behaviours. Parental support and older age at drug initiation appeared to be protective factors.

**Discussion:**

The findings characterise the correlates of suicidal behaviours among school-going Lebanese adolescents and determine the attributes of the risk group susceptible to engaging in suicide attempts. Future interventions and policies should consider these attributes when monitoring target groups, particularly those with the alarming behaviours identified in this study. In addition, awareness campaigns that engage all stakeholders, particularly parents, should be prioritised by the authorities.

## Introduction

Suicide is widely documented as a serious public health problem and is often underestimated, particularly in countries where it is considered a crime. Nonetheless, it is a significant contributor to mortality in most countries worldwide [1]. Globally, it was estimated that over 700,000 people died from suicide in 2019, with 77% of the deaths occurring in low- and middle-income countries [2]. Furthermore, 88% of adolescent suicides occurred in low- and middle-income countries [2]. Vulnerable groups, such as young people, are highly susceptible to experiencing suicidal ideation and attempting suicide. Suicide among young people aged 10–19 years is a matter of global concern, accounting for approximately one-third of the global suicide incidence, and it is the fourth leading cause of death among these age groups [2]. Adolescent suicide has been regarded as a serious phenomenon that affects the well-being of the youth over previous decades. Hence, suicide prevention has become imperative to save numerous young people’s lives, thereby underscoring the importance of identifying relevant risk and protective factors to inform and develop preventive strategies to reduce suicide among adolescents.

Adolescents undergo rapid physical, cognitive, and psychosocial transitions [3]. During this period, they are vulnerable to a range of risky behaviours, including but not limited to physical fights, bullying, substance use, and risky sexual behaviours [4, 5]. Moreover, feelings of loneliness are particularly prevalent during adolescence [6]. Previous research has identified several risk factors linked to suicidal ideation and behaviour, such as loneliness [7–9], physical fights [9–11], bullying victimization [9–14], and food insecurity [15]. Additionally, adolescents who engage in substance use, such as cigarettes, marijuana, or alcohol, have exhibited associations with suicidal ideation and attempts [9, 10, 14]. In terms of protective factors, high levels of parental support [16] as well as parental understanding and monitoring [17] are negatively associated with suicide.

Lebanon is a country located in the Middle East, neighbouring Syria to the north and Israel to the south, and has suffered from conflicts or wars in the previous decades. It was reclassified from an upper-middle income country to a low- or middle-income country because of the economic and financial crises in the previous three years [18]. Similar to most countries affected by war in the region, suicidal ideation among adolescents is a frequent phenomenon. A recent survey in 2020 reported an estimated 28.9% rate of suicidal ideation among Lebanese adolescents aged 14–17 years [19]; however, the proportion of those who consequently attempted suicide was not reported. In addition, suicide is a religious taboo in Lebanon [20], preventing people from seeking support, which often leads to tragic and lethal suicide attempts and causes enormous burdens and disabilities, particularly at an early age [20–22]. This can be prevented by identifying the risk groups and ensuring early interventions.

Given this background, more studies are needed to assess the prevalence and identify the determinants of suicidal behaviours among Lebanese adolescents. This study aims to investigate the prevalence and association of suicidal behaviours with physical/psychological harm and substance use in a representative sample of adolescents in Grades 7–12 in Lebanon. It will provide a deeper understanding of suicidal behaviours and their correlates in Lebanese adolescents and could be beneficial to not only Lebanon but also other Eastern Mediterranean regions to ideate future suicide intervention programmes.

## Methods

### Study setting

This study entails secondary data analysis using data from the 2017 Lebanon Global School-based Student Health Survey (GSHS). The first and second rounds of the GSHS were conducted in 2005 and 2011 among students in Grades 7–9; high school students in Grades 10–12 were also included to monitor the prevalence of important health risk behaviours and protective factors among school-going adolescents in Grades 7–12 at the national level.

### Sampling and response rate

For the 2017 Lebanon GSHS, a two-stage cluster sample design was employed by the Centres for Disease Control and Prevention (CDC) to ensure a representative sample of students in Grades 7–12. The first stage involved selecting schools, with all schools encompassing Grades 7–12 being included in the sampling framework provided by the Ministry of Education and Higher Education, with the majority of students in Grades 7–12 was included in the sampling frame. The CDC used systematic equal-probability sampling with a random start to select classes from each participating school. All students in the sampled classrooms were eligible to participate in the GSHS. Of the 64 selected schools, 56 participated in the survey, resulting in a school response rate of 88%. In addition, 5717 of the 6152 selected students completed the survey. Therefore, the student response rate was 93%, and the overall response rate was 82% (88% × 93% = 82%). A weighting factor was applied to each student record to adjust for non-response and varying selection probabilities, and more details about the weighting factor calculation are provided in the final report of the Lebanon GSHS 2017 [23].

### Data collection

A standard GSHS questionnaire was administered to the students during a regular class period. The questionnaire included questions related to the following main themes: demographics (age, sex, and grade), anthropometric characteristics (weight and height), dietary habits, personal and oral hygiene, mental health, violence and unintentional injuries, bullying, substance use (tobacco, alcohol, and illegal drugs), sexual and reproductive health, parental support, and physical activity. Before the survey, all the students were informed of the content of the survey, their privacy and rights, and their voluntary participation.

### Outcome variables

*Suicidal ideation* was assessed by the question, ‘During the past 12 months have you ever seriously considered attempting suicide?’ The answers were binary (yes = 1; no = 0).

*Suicide attempt* was assessed by the question. ‘During the past 12 months, how many times did you actually attempt suicide?’ The answers were coded as binary variable (yes = 1; no = 0). However, in the multivariate analysis, suicide attempts were assessed only for those who had suicidal ideation. By limiting the analysis to individuals with suicidal ideation, we aimed to capture the population at the highest risk of suicide attempts [24]. This approach allowed for a focussed examination of the factors associated with transitioning from suicidal ideation to actual suicide attempts, thereby formulating preventive strategies that can effectively intervene during this critical period [25].

### Explanatory variables

#### Demographic variables

Age (11–18 years), sex (male or female), grades (7–12).

#### Physical and psychological harms

*Felt lonely* was assessed by the question, ‘During the past 12 months, how often have you felt lonely?’, and the answers were coded as 1 (never/rarely), 2 (sometimes), 3 (most of the time), and 4 (always).

*Felt worried* was assessed by the question, ‘During the past 12 months, how often have you been so worried about something that you could not sleep at night?’, and the answers were coded as 1 (no), 2 (1 time), and 3 (≥ 2 times).*Physically attacked* was assessed by the question, ‘During the past 12 months, how many times were you physically attacked?’, and the answers were coded as 1 (no), 2 (1 time), and 3 (≥ 2 times).

*Physical fight* was assessed by the question, ‘During the past 12 months, how many times were you in a physical fight?’, and the answers were coded as 1 (no), 2 (1 time), and 3 (≥ 2 times).

*Type of bullying* was coded as 1 (not bullied), 2 (kicked, pushed, or shoved), 3 (made fun of based on the race), 4 (made fun of because of religion), 5 (made fun of about sex), 6 (left out of activities), 7 (made fun of body), and 8 (some other way).

*Missed school* was assessed by the question, ‘During the past 30 days, on how many days did you miss classes or school without permission?’, and the answers were coded as 1 (no), 2 (1–2 days), and 3 (≥ 3 days).

*Parental support* was assessed by the question, ‘During the past 30 days, how often did your parents or guardians understand your problems and worries?’, and the answers were coded as 1 (never/rarely), 2 (sometimes), 3 (most of the time), and 4 (always).

*Food insecurity* was assessed by the question, ‘During the past 30 days, how often did you go hungry because there was not enough food in your home?’, and the answers were coded as 1 (never/rarely), 2 (sometimes), and 3 (most of the time/always).

#### Substance use-related variables

*Ever/current marijuana/amphetamine use* was coded as 0 (no) and 1 (yes).

*Age at drug/cigarette/alcohol*, as previous studies had highlighted the importance of early initiation into drug use and emphasised ages 10–13 as a time of heightened vulnerability for mental issues [26, 27], this variable was coded as 1 (≤ 9), 2 (10–13), and 3 (≥ 14).

*Current use of cigarette/other tobacco products/alcohol drinking* was coded as 0 (no), 1 (1–5 days/month), and 3 (≥ 6 days/month).

*Quantity of alcohol* was evaluated based on the question, ‘During the past 30 days, on the days you drank alcohol, how many drinks did you usually drink per day?’, and the answers were coded as 0 (no), 1 (≤ 1/day), and 3 (≥ 2/day).

*Got drunk/troubled drunk* was assessed by the questions, ‘During your life, how many times did you drink so much alcohol that you were really drunk?’ and ‘During your life, how many times have you got into trouble with your family or friends, missed school, or got into fights, as a result of drinking alcohol?’ The answers were coded as 0 (no) and 1 (yes).

## Statistical analysis

The descriptive analysis demonstrated the distribution of sample characteristics, physical and psychological harm, substance use-related variables, and suicidal ideation and attempts among Lebanese adolescents in Grades 7–12. Logistic regression analysis, while adjusting for age and sex, was used to examine the likelihood of suicidal ideation and subsequent suicide attempts across the explanatory variables in this study, and the results were reported as adjusted odds ratios (aOR) and 95% confidence intervals (95%CI). The approach of estimating the independent effects of the study variables on suicidal ideation and attempts, regardless of the potential confounding effects of age and sex, can identify the individual contributions of each factor to the outcome variable, thereby allowing for targeted school-based interventions and preventive strategies for all students in Grades 7–12, while providing valuable information for policymakers. Sampling design and weights were applied by defining the survey strata, primary sampling unit, and weight using in STATA 17. The statistical significance level was set at p < 0.05.

## Results

### The prevalence of suicidal ideation and suicide attempt among Lebanese adolescents in Grades 7–12

From Table 1, 9.74% of Lebanese adolescents in Grades 7–12 attempted suicide in the previous year. In terms of suicidal ideation, an estimated 13.45% of Lebanese adolescents in Grades 7–12 seriously considered suicide in the previous year, whereof 46.82% attempted suicide.

**Table 1:** Prevalence of suicidal ideation and attempt among Lebanese adolescents in 7th−12th grades in the past year

	N (weighted %)
Suicide attempt	568 (9.74)
Suicidal ideation	765 (13.45)
Suicide attempt^a^	360 (46.82)
Total	5692 (100)

^a^Among those who had suicidal ideation

### Sample characteristics and their association with suicidal ideation/suicide attempt in Lebanese adolescents in Grades 7–12

According to Table 2, 5692 adolescents in Grades 7–12 were included in the present study. The mean age of participants was 14.60 (± 0.14) years old, ranging from 11–18 years. There were comparatively similar weighted proportions of adolescents in each age group; however, a higher number of students were recruited from the age group ≥ 16. Although those in age groups 14–15 (aOR = 1.33; 95%CI : 1.11–1.59) and ≥ 16 years (aOR = 1.37; 95%CI : 1.13–1.68) were more likely to consider suicide than those in the age group 11–13 in the previous year, there was no significant association between age group and those who actually attempted suicide after consideration. There was a relatively equal weighted proportion of males (46.81%) and females (53.19%) in this study, and females were more likely to attempt suicide (aOR = 1.35; 95%CI : 1.02–1.78). Approximately half of the adolescents were either in Grade 7 (23.52%) or 8 (20.04%), and there was no association between grade and suicide.

**Table 2:** Demographic characteristics and suicidal ideation/attempt among Lebanese adolescents in 7th−12th grades

Variable	N (weighted %)	Suicidal ideation	Suicide attempt^a^
		aOR (95%)	aOR (95%)
Age group			
11–13	1543 (31.29)	Ref.	
14–15	1878 (34.98)	1.33 (1.11–1.59)	1.20 (0.66–2.21)
≥ 16	2271 (33.73)	1.37 (1.13–1.68)	1.29 (0.74–2.23)
Sex			
Male	2330 (46.81)	Ref.	Ref.
Female	3370 (53.19)	1.12 (0.97–1.30)	1.35 (1.02–1.78)
Grade			
7th	1247 (23.52)	Ref.	Ref.
8th	1125 (20.04)	1.36 (0.94–1.99)	0.99 (0.55–1.81)
9th	783 (17.08)	1.33 (0.87–2.04)	1.80 (0.80–4.06)
10th	959 (15.00)	1.20 (0.83–1.73)	1.57 (0.78–3.13)
11th	708 (12.90)	1.13 (0.80–1.58)	1.01 (0.49–2.07)
12th	859 (11.46)	1.11 (0.76–1.60)	0.72 (0.33–1.57)

^a^Among those who have suicidal ideation

### Physical and psychological harms and their association with suicidal ideation/suicide attempt in Lebanese adolescents in Grade 7–12

Almost one-third of the adolescents in Grades 7–12 grades reported feeling lonely (36.81%) or worried (31.12%) for at least some time in the previous 12 months. Those who sometimes, most of the time, and always felt lonely in the previous 12 months were, respectively, 2.26, 5.67, and 12.73 times more likely to consider suicide as compared to those who never or rarely felt lonely. Similarly, those who sometimes felt worried tended to consider suicide more frequently (Table 3). Among those who considered suicide, those who always felt lonely (aOR = 3.84; 95%CI : 2.32–6.37) or always felt worried (aOR = 3.76; 95%CI : 2.32–6.09) were more likely to attempt suicide than those who never or rarely had these feelings.

**Table 3:** Physical/psychological harms and suicidal ideation/ suicide attempt among Lebanese adolescents in 7th−12th grades

Variable	N (weighted %)	Suicidal ideation	Suicide attempt^a^
		OR (95%)^b^	OR (95%)^b^
Felt lonely (last 12 months)			
Never/rarely	3457 (63.19)	Ref.	Ref.
Sometimes	1368 (23.16)	2.26 (1.71–2.99)	1.08 (0.68–1.70)
Most of the time	574 (9.19)	5.67 (3.82–8.43)	0.99 (0.60–1.66)
Always	265 (4.46)	12.73 (8.23–19.68)	3.84 (2.32–6.37)
Felt worried (last 12 months)			
Never/rarely	3750 (68.88)	Ref.	Ref.
Sometimes	1105 (19.23)	1.75 (1.35–2.28)	1.36 (0.86–2.15)
Most of the time	484 (7.81)	4.26 (2.80–6.48)	2.42 (1.31–4.48)
Always	253 (4.08)	9.74 (7.01–13.53)	3.76 (2.32–6.09)
Physically attacked (last 12 months)			
No	4561 (79.48)	Ref.	Ref.
1 time	523 (9.38)	1.89 (1.53–2.33)	1.55 (0.93–2.59)
≥ 2 times	606 (11.13)	2.83 (2.32–3.47)	1.70 (1.13–2.55)
Physical fight (last 12 months)			
No	3684 (61.73)	Ref.	Ref.
1 time	814 (15.63)	1.24 (0.97–1.59)	1.43 (0.75–2.71)
≥ 2 times	1147 (22.64)	2.17 (1.60–2.93)	2.20 (1.43–3.38)
Bullying history (last 30 days)			
No	4651 (83.52)	Ref.	Ref.
1–2 times	509 (10.46)	2.39 (1.72–3.34)	1.36 (0.77–2.40)
≥ 3 times	301 (6.02)	4.06 (3.05–5.40)	1.51 (0.80–2.91)
Type of Bullying			
Not bullied	4765 (86.80)	Ref.	Ref.
Kicked, pushed, or shoved	149 (3.27)	2.76 (1.74–4.39)	3.96 (1.52–10.31)
Made fun of the race	68 (1.34)	2.86 (1.60–5.12)	0.77 (0.23–2.60)
Made fun because of religion	42 (0.74)	5.17 (2.31–11.56)	1.73 (0.28–10.80)
Made fun of about sex	91 (1.93)	2.58 (1.58–4.23)	5.39 (1.74–16.59)
Left out of activities	21 (0.44)	3.41 (1.25–9.31)	2.88 (0.62–13.36)
Made fun of body	58 (1.07)	4.68 (1.99–11.03)	1.53 (0.75–3.08)
Some other way	229 (4.41)	1.98 (1.27–3.09)	1.29 (0.73–2.28)
Missed school (last 30 days)			
No	4406 (83.57)	Ref.	Ref.
1–2 days	610 (11.09)	1.35 (1.10–1.66)	1.28 (0.65–2.51)
≥ 3 days	288 (5.34)	2.78 (1.78–4.34)	0.79 (0.44–1.39)
Parental support			
Never/rarely	1307 (26.95)	Ref.	Ref.
Sometimes	958 (20.44)	0.58 (0.38–0.88)	0.84 (0.49–1.46)
Most of the time	956 (21.36)	0.38 (0.27–0.53)	0.96 (0.50–1.83)
Always	1505 (31.25)	0.46 (0.34–0.61)	1.34 (0.68–2.64)
Food insecurity (last month)			
Never/rarely	5019 (87.91)	Ref.	Ref.
Sometimes	502 (8.75)	1.58 (1.27–1.97)	1.60 (1.01–2.52)
Most of the time/ Always	163 (3.34)	3.32 (1.96–5.65)	2.71 (1.24–5.91)

^a^Among those who have suicidal ideation

^b^Adjusted for age and sex

Approximately one-fifth of the adolescents in this study had been physically attacked by someone, and more than one third had engaged in a physical fight with others in the previous year. It appeared that the likelihood of suicidal ideation and subsequent suicide attempts was significantly higher among those who had been physically attacked or engaged in a physical fight more than twice in the past 12 months compared to those who had not; additionally, the more there were such experiences, the greater the likelihood of suicidal behaviours observed (Table 3).

An estimated 16.48% of participants were bullied at least once in the previous 30 days, and higher levels of suicidal ideation were observed among those who were bullied ≥ 3 times (aOR = 4.06; 95%CI : 3.05–5.40) and 1–2 times (aOR = 2.39; 95%CI : 1.72–3.34) than those who were not. No association was observed between this variable and suicide attempt. The most common types of bullying were being kicked, pushed, or shoved (3.27%); made fun of based on race (1.34%); and made fun of based on body (1.07%). An estimated 16.43% had missed school at least once in the previous month for no reason. Although those who were being kicked/pushed/shoved (aOR = 2.76; 95%CI : 1.74–4.39) or bullied because of their race (aOR = 2.86; 95%CI : 1.60–5.12), religion (aOR = 5.17; 95%CI : 2.31–11.56), sex (aOR = 2.58; 95%CI : 1.58–4.23), and body shape (aOR = 4.68; 95%CI : 1.99–11.03) or those who were left out of activities (aOR = 3.41; 95%CI : 1.25–9.31) were more likely to consider suicide, only those who were being kicked/pushed/shoved (aOR = 3.96; 95%CI : 1.52–10.31) because of sex (aOR = 5.39; 95%CI : 1.74–16.59) tended to attempt suicide afterwards.

Approximately 16.43% of adolescents skipped school at least once in the previous month for no reason and the odds of suicidal ideation were 1.35 and 2.78 times higher among those who skipped 1–2 days and ≥ 3 days, respectively, compared to those who did not.

No association was observed between missing school and suicide attempts. Approximately 73% of the adolescents in the study reported having parental support (understanding of worries and problems) sometimes/most of the time/always in the last 30 days, and they were less likely to consider suicide than those who did not receive such support. However, such a relationship was not observed in subsequent suicide attempts (Table 3). Only 12.09% reported going hungry because of inadequate food at home in the last 30 days, and it appeared that the likelihood of suicidal ideation and subsequent attempts increased considerably with food insecurity (Table 3).

### Substance use and its association with suicidal ideation/suicide attempt in Lebanese adolescents in Grades 7–12

As shown in Table 4, 141 (2.39%) and 90 (1.52%) adolescents reported, marijuana and amphetamine use at least once in their lifetime, respectively, and 109 (2.01%) reported current marijuana use. There was a significant association between ever (aOR = 6.09; 95%CI : 4.09–9.08) or current (aOR = 6.00; 95%CI : 2.94–12.24) marijuana and ever amphetamine (aOR = 4.16; 95%CI : 2.50–6.91) use with suicidal ideation but not with subsequent suicide attempts, except for marijuana use (aOR = 2.66; 95%CI : 1.24–5.67). Almost half of the sample (49.82%) initiated drug use at age ≤ 9 years and approximately two-thirds at age < 14 years; however, no significant association was found between age at drug initiation and suicidal behaviours. Nearly 13% reported current cigarette use, whereas 31.30% used other tobacco products (e.g., waterpipes and narghiles).

**Table 4:** Substance use and suicidal ideation/suicide attempt among Lebanese adolescents in 7th−12th grades

Variable	N (weighted %)	Suicidal ideation	Suicide attempt^a^
		OR (95%)^b^	OR (95%)^b^
Ever marijuana use			
No	5307 (97.61)	Ref.	Ref.
Yes	141 (2.39)	6.09 (4.09–9.08)	2.66 (1.24–5.67)
Current marijuana use			
No	5327 (97.99)	Ref.	Ref.
Yes	109 (2.01)	6.00 (2.94–12.24)	1.97 (0.91–4.25)
Ever amphetamine use			
No	5161 (98.48)	Ref.	Ref.
Yes	90 (1.52)	4.16 (2.50–6.91)	2.24 (0.79–6.29)
Age at drug initiation (years)			
≤ 9	100 (49.82)	Ref.	Ref.
10–13	43 (23.67)	0.59 (0.21–1.66)	2.31 (0.61–8.80)
≥ 14	49 (26.51)	1.14 (0.48–2.70)	0.33 (0.02–5.09)
Current cigarette use			
No	4847 (86.89)	Ref.	Ref.
1–5 days/month	401 (7.60)	2.87 (2.13–3.87)	1.50 (0.91–2.46)
≥ 6 days/month	304 (5.51)	3.38 (2.14–5.33)	3.71 (2.15–6.39)
Current use of other tobacco products			
No	3781 (68.70)	Ref.	Ref.
1–5 days/month	1142 (19.68)	2.25 (1.73–2.92)	1.20 (0.71–2.06)
≥ 6 days/month	699 (11.63)	3.83 (2.95–4.96)	3.06 (1.91–4.90)
Age at cigarette initiation (years)			
≤ 9	322 (24.36)	Ref.	Ref.
10–13	504 (41.20)	0.80 (0.49–1.32)	1.99 (0.70–5.68)
≥ 14	484 (34.45)	0.79 (0.48–1.27)	2.53 (1.02–6.26)
Drink alcohol			
No	4664 (82.53)	Ref.	Ref.
1–5 days/month	566 (12.31)	1.56 (1.10–2.22)	1.90 (1.15–3.14)
≥ 6 days/month	250 (5.16)	2.55 (1.57–4.17)	2.36 (1.27–4.39)
Quantity of alcohol (at each event)			
No	4673 (81.80)	Ref.	Ref.
≤ 1/day	453 (9.99)	1.44 (1.02–2.04)	1.72 (1.01–2.91)
≥ 2/day	399 (8.21)	2.22 (1.35–3.67)	2.21 (1.26–3.88)
Got drunk (lifetime)			
No	4884 (82.89)	Ref.	Ref.
Yes	824 (17.11)	1.92 (1.22–3.3)	1.56 (0.93–2.62)
Troubled drunk			
No	4316 (71.95)	Ref.	Ref.
Yes	1392 (28.05)	1.38 (1.07–1.77)	0.76 (0.52–1.12)
Age at first drink (years)			
≤ 9	320 (19.29)	Ref.	Ref.
10–13	481 (30.50)	0.59 (0.34–1.00)	0.66 (0.32–1.38)
≥ 14	327 (50.21)	0.50 (0.29–0.84)	0.83 (0.33–2.06)

^a^Among those who have suicide ideation

^b^Adjusted for age and sex

Higher suicidal ideation levels were observed among smokers regardless of the frequency of smoking; however, only those who smoked ≥ 6 days/month tended to have a higher likelihood of suicide attempts for cigarette smoke (aOR = 3.71; 95%CI : 2.15–6.39) and other tobacco products (aOR = 3.06; 95%CI : 1.91–4.90). Approximately one-fifth of the participants started smoking cigarettes before the age of 9 years; however, no significant relationship was observed between this variable and suicidal behaviours.

An estimated 17.47% of the adolescents had a history of drinking alcohol at least once a month, and only 8.21% had ≥ 2 drinks per day on occasion. Alcohol consumption was associated with a higher likelihood of suicidal ideation and attempt, with higher frequency and quantity being strong predictors of a higher risk of suicidal behaviours (Table 4). Approximately 20% of them started drinking alcohol at age ≤ 9 and higher ages of drinking initiation; particularly, adolescents at age ≥ 14 (aOR = 0.50; 95%CI : 0.29–0.84) was associated with significantly lower odds of suicidal ideation. While 17.11% reported getting drunk in their lifetime, approximately 28% had troubled drinking experiences. Although getting drunk and troubled drinking behaviours appeared to increase the likelihood of suicidal ideation, they had no effect on suicide attempts (Table 4).

## Discussion

This study sheds light on the prevalence of suicidal behaviours and their correlates in Lebanese adolescents, highlighting the great concern regarding suicide within the young population. Previous studies in Lebanon have emphasised suicide among these age groups as an important issue to be addressed [28, 29]; however, a thorough report on the causes of such dangerous behaviours in Lebanese teenagers has not yet been published in recent years. This study provides comprehensive information using data from a nationally representative sample of adolescents in Grades 7–12 in Lebanon. The findings showed that approximately 13.45% of Lebanese adolescents in Grades 7–12, particularly females, had considered suicide at least once in the previous 12 months. More importantly, almost half of those who considered suicide had attempted it at least once in the previous 12 months. Regarding psychological and physical harm, those who experienced loneliness or worry frequently in the previous year, were involved in physical fights or assaults in the previous year, had been verbally or physically bullied in the previous month, had missed more school days, and experienced food insecurity were more likely to engage in suicidal behaviours. However, parental support appeared to be a protective factor. Concerning the impact of substance abuse, in general, those with a history of substance abuse (marijuana, amphetamine, alcohol, or tobacco products) were more likely to engage in suicidal behaviours. Older age at initiation appeared to be a protective factor, especially regarding alcohol consumption behaviours.

In line with our results, previous studies have reported higher levels of suicidal behaviours among adolescents who have been exposed to physical violence or engaged in physical fights [9, 10]. Similarly, a higher proportion of both male and female suicide attempters reported fighting than those who had not attempted suicide [11]. Research has also shown an association between bullying involvement and suicidal thoughts and behaviours [29–32]. However, these studies did not specify the types of bullying involved. It is important to note that discussing bullying as the sole cause of suicide is unhelpful and can be potentially harmful. According to our research, adolescents who experience physical or verbal bullying based on gender, physical appearance, race, or religion are more likely to engage in suicidal conduct. Therefore, more research is advised to characterise the stigmatised races and religions in Lebanese schools to identify the minority groups that are the most at risk and provide them with more support in educational initiatives. This study found an association between suicidal ideation and school absenteeism, which was consistent with previous studies [33, 34]. However, no such relationship was observed in the subsequent suicide attempts. Therefore, further research is required to examine the underlying mechanisms of this association. With regard to food insecurity among adolescents, a cross-sectional study of 179,771 adolescents from 9 high-income, 31 middle-income, and 4 low-income countries found an association between food insecurity (hunger) and suicide attempts [35]. A study conducted among US high school students [36] also demonstrated an association between food insecurity and both suicidal ideation and suicide attempts, which is consistent with our findings. Food insecurity has recently become a major issue in Lebanon, with 42% of the country’s population facing it by 2023[37]. This issue affects not only the health and well-being of children but also their education. Children from food-insecure households are at risk of dropping out of school or not enrolling at all because of the pressure to become the main breadwinners of the family, all of which are associated with poorer mental health and consequent risky behaviours [38]. Therefore, urgent action is required to prevent student malnutrition and improve access to food in schools.

Adolescents are vulnerable in terms of substance use and suicide. A higher risk of suicide was observed among adolescents with a history of current substance use, and a significant proportion of suicides involved the use of substances such as marijuana, amphetamine, and alcohol [9, 10, 14, 39, 40]. Substance use disorders are associated with an almost 10–14 times greater risk of suicide than the general population, with alcohol use disorders being the leading cause of deaths related to substance use across all age groups, consistent with the findings of the present study [41]. Additionally, in our study, we find higher suicidal behaviours among those who currently use cigarettes or tobacco products, which has been documented in previous studies [10, 40], but not completely in the adolescent population or certainly among Lebanese adolescents. Moreover, there are limited data on the specific prevalence of suicide among adolescents who use specific substances such as marijuana, amphetamine, alcohol, and cigarettes across countries [42]. However, this study reported on the risk of suicidal behaviours specific to each substance use disorder among school-going Lebanese adolescents using the latest data from a national survey, which could have important policy implications for Lebanese schools to prevent further harm and risky behaviours. This could be achieved by taking concrete steps to promote connectedness among the youth [43], developing initiatives that could address children’s development across different stages and provide effective services at the community level, engaging parents and school administrations, and preventing drug abuse among children and adolescents.

In this study, two protective factors against suicidal behaviours were observed. First, the supportive behaviour of parents appeared to be associated with lower suicidal ideation but not suicide attempts, and second, older age regarding the initiation of substance use, particularly alcohol and drugs. Previous studies have shown that the perception of understanding and supportive attitudes of parents can have a protective effect on adolescents’ suicidal behaviour, with the positive influence being slightly stronger in girls than in boys [44]. In addition, attentive and supportive parental behaviours can prevent suicide among adolescents who struggle with sadness, anxiety, and depression [45, 46]. Therefore, such behaviours should be consistently encouraged in schools by engaging parents in educational activities. Evidence also implies an increased risk of suicidal ideation and attempts as well as early initiation of substance use [47, 48], which is also in line with our findings. In Lebanon, the common age of onset of substance use is 14–15 years, with alcohol being the most commonly used substance among adolescents [49]. Several strategies have been implemented by the International Society of Substance Use Professionals to prevent substance use in Lebanese schools [50]; however, the success of such programmes has not yet been assessed and should be at direction for future research.

Although this study is the first and latest report of suicidal ideation and attempts and their correlates among a nationally representative sample of adolescents in Grades 7–12 in Lebanon, we are unable to determine the factors that lead to a successful suicide attempt because conclusions are often drawn from suicidal ideation and preparation. In addition, the cross-sectional design of the study merely enabled us to identify connections rather than causal relationships; thus, further prospective studies investigating factors linked to successful suicide are recommended, even though they could be challenging. Underreporting by excluding teenagers who are not enrolled in school and who lack relevant demographic information such as socioeconomic background, religion, and family dynamics is another drawback. However, because the data were drawn from the WHO’s Global Schools Health Survey, the study’s large sample size ensured the diversity and representativeness of the data.

## Conclusion

The findings characterized the correlates of suicidal behaviours among school-going Lebanese adolescents and determine the attributes of the risk group susceptible to engage in suicide attempts. Future interventions and policies should consider these attributes when monitoring target groups, particularly those with the alarming behaviours identified in this study. In addition, awareness campaigns that engage all stakeholders, particularly parents, should be prioritised by the authorities.

## Data Availability

The GSHS 2017 is a public dataset available at WHO official website (https://extranet.who.int/ncdsmicrodata/index.php/catalog/645/study-description) and could be downloaded upon a reasonable request by a registered user and with permission from the WHO website.
